# Clinical implications of cardiovascular outcome trials in type 2 diabetes

**DOI:** 10.1007/s00059-019-4789-4

**Published:** 2019-02-25

**Authors:** L. G. Mellbin, A. Wang, L. Rydén

**Affiliations:** 1 Cardiology Unit, Department of Medicine K2, Karolinska Institutet, Stockholm, Sweden; 2 Heart and Vascular Theme, Karolinska University Hospital, 17176 Stockholm, Sweden

**Keywords:** Diabetes mellitus, adult-onset, Cardiac diseases, Sodium–glucose cotransporter 2 inhibitors, Dipeptidyl-peptidase 4 inhibitors, Glucagon-like peptide 1 receptor agonists, Diabetes mellitus mit Beginn im Erwachsenenalter, Herzerkrankungen, Natrium-Glukose-Kotransporter-2-Hemmer, Dipeptidylpeptidase-4-Hemmer, Glucagon-like-Peptide-1-Rezeptoragonisten

## Abstract

Cardiovascular disease (CVD) is the main reason for premature death in patients with type 2 diabetes. Hyperglycemia, the hallmark of diabetes, has long been considered the link between diabetes and CVD, and many trials focused on preventing CVD manifestations by means of tight glucose control. However, diabetes is a multifactorial disease in which, e. g., insulin resistance, endothelial dysfunction, and factors such as hypertension and dyslipidemia contribute. Thus, treatment needs to be multifactorial and take cardiovascular aspects into account. Newer classes of drugs, originally launched for glucose lowering, among them dipeptidyl-peptidase (DPP)-4 inhibitors, sodium–glucose cotransporter (SGLT)-2 inhibitors, and glucagon-like peptide (GLP)-1 receptor agonists, have been studied in large cardiovascular outcome trials (CVOT). Several SGLT-2 inhibitors and GLP-1 receptor agonists are associated with a reduction of cardiovascular events (cardiovascular death, nonfatal myocardial infarction, and nonfatal stroke). Although the mechanisms behind the effects are not fully understood, an important reason for the benefits of SGLT-2 inhibitors seems be a reduction in heart failure, while GLP-1 receptor agonists may retard the development of the atherosclerotic vascular disease or may be effective by stabilizing plaques. The outcomes of these studies have been taken into account in recently issued guidelines and an important task for diabetologists, cardiologists, and general practitioners is to incorporate the findings of these trials into clinical practice.

## Zusammenfassung

Kardiovaskuläre Erkrankungen (CVD) sind der Hauptgrund für einen vorzeitigen Tod bei Patienten mit Typ-2-Diabetes. Die Hyperglykämie, Kennzeichen des Diabetes mellitus, wurde lange als Bindeglied zwischen Diabetes und CVD angesehen, und viele Studien legten den Fokus auf die Prävention von CVD mittels strenger Glukoseeinstellung. Diabetes ist jedoch eine multifaktorielle Erkrankung, zu der z. B. Insulinresistenz, endotheliale Funktionsstörungen sowie Faktoren wie Hypertonie und Fettstoffwechselstörungen beitragen. Daher muss die Behandlung multifaktoriell sein und kardiovaskuläre Aspekte berücksichtigen. Neuere Substanzklassen bei Medikamenten, die ursprünglich zur Glukosesenkung eingeführt worden waren, darunter Dipeptidylpeptidase(DPP)-4-Inhibitoren, Natrium-Glukose-Kotransporter(SGLT)-2-Inhibitoren und Glucagon-like-Peptide(GLP)-1-Rezeptoragonisten, sind in großen kardiovaskulären Endpunktstudien (CVOT) untersucht worden. Verschiedene SGLT-2-Inhibitoren und GLP-1-Rezeptoragonisten gehen mit einer Verminderung kardiovaskulärer Ereignisse einher (kardiovaskulär bedingter Tod, nichtletaler Myokardinfarkt und nichtletaler Schlaganfall). Auch wenn die den Auswirkungen zugrunde liegenden Mechanismen nicht vollständig verstanden worden sind, scheint ein wichtiger Grund für den Nutzen von SGLT-2-Inhibitoren in einem selteneren Auftreten der Herzinsuffizienz zu bestehen, während GLP-1-Rezeptoragonisten möglicherweise die Entstehung atherosklerotischer Veränderungen der Gefäße verzögern oder durch Stabilisierung von Plaques wirken. Die Ergebnisse dieser Studien wurden in aktuell herausgegebenen Leitlinien berücksichtigt; eine wichtige Aufgabe für Diabetologen, Kardiologen und Allgemeinmediziner ist es, die Erkenntnisse aus diesen Studien in den klinischen Praxisalltag einzubringen.

## Diabetes and cardiovascular disease

### Epidemiology

Cardiovascular disease (CVD) is the main reason for premature death in patients with type 2 diabetes mellitus (T2DM; [1]). The already large number of people living with established T2DM—425 million worldwide in the age groups 20–79 years in 2017—is estimated to increase to 629 million in 2045. Thus, diabetes-related CVD currently constitutes a considerable global health problem and, if not counteracted, will become an even greater problem in time. This also contributes to a large part, presently 12%, of global health expenditure [1].

In the middle of the twentieth century, it was acknowledged that people with T2DM are prone to developing macrovascular complications such as myocardial infarction and that their survival is affected [2]. Although the prognosis after an acute myocardial infarction has improved in the past few decades, survival is still considerably shorter in patients with diabetes than those without [3, 4].

In addition to the increased risk of coronary artery disease, the association between diabetes and other cardiovascular manifestations such as heart failure, arrhythmias, and peripheral artery disease has gained increasing interest. For example, a large prospective cohort study (*n* = 1,921,260, of whom 34,198 [1.8%] had T2DM) based on the CVD research using the Clinical Disease Research Using Linked Bespoke Studies and Electronic Health Records (CALIBER) program showed that during a median follow-up of 5.5 years, peripheral arterial disease and heart failure were the first presentation of cardiovascular events in, respectively, 16.2% and 14.1% of the individuals with T2DM [5]. In addition, patients with the combination of heart failure and T2DM have a poor prognosis [6]. It is important that the wide array of manifestations of CVD in people with T2DM is taken into consideration when assessing cardiovascular risk and when setting up treatment plans. It is also of importance for the design and interpretation of cardiovascular outcome studies.

Hyperglycemia, the hallmark of T2DM, has been considered as the link between diabetes and CVD since this association was detected following the institution of insulin treatment in the 1920s [7, 8]. The connection between high blood glucose and CVD has been confirmed in several populations [9, 10]. Not surprisingly, a majority of early pharmacological attempts to reduce such complications have focused on strict glycemic control.

## Cardiovascular outcome trials

### Strict glycemic control

Until recently, the majority of the trials on people with T2DM focused on glucose lowering either in the context of the effect of different drugs such as insulin and sulfonylureas or assessing different treatment strategies (e. g., intensive vs. less intensive).

The UK Prospective Diabetes Study (UKPDS) study, starting in 1977, comprised patients with newly detected T2DM. They were randomized to intensified glycemic control that was compared with conventional care. After 10 years, intensive glucose control by either sulfonylureas or insulin decreased the risk of microvascular but not of macrovascular complications [11]. In a post-trial follow-up a further 10 years later, and despite an early loss of glycemic differences between the study groups, a reduced risk for myocardial infarction (15%, *p* = 0.01) and death from any cause (13%, *p* = 0.007) emerged as more events occurred [12]. Furthermore, in the UKPDS 34 study [13], 342 overweight individuals were randomized to receive metformin or conventional care. A significant reduction in the occurrence of myocardial infarction was seen in the metformin arm at the initial follow-up already. This was sustained in the post-trial follow-up for myocardial infarction (33%, *p* = 0.005), and death from any cause (27%, *p* = 0.002; [12]). An important aspect to consider when interpreting these findings is that by contemporary means, the UKPDS patients received only to a very small extent effective lipid- (0.3%) and blood-pressure lowering drugs (12%) [11].

It is somewhat disappointing that the beneficial effect of intensive glucose lowering could not be verified in three trials published in 2009: the Action in Diabetes and Vascular Disease (ADVANCE; [14]), the Action to Control Cardiovascular Risk in Diabetes (ACCORD; [15]), and the Veterans Administration Diabetes Trial (VADT; [16]). These trials recruited patients suffering from diabetes for 8–11.5 years, of whom 32–40% had CVD. None of these studies, with a follow-up of 3.5–5.6 years, confirmed that intensive glucose lowering improved cardiovascular prognosis [17]. In fact, the ACCORD trial was prematurely stopped owing to an increased cardiovascular mortality rate among intensively managed subjects (HR: 1.35; CI 1.04–1.76; *p* = 0.02; [15]). It was suggested that this was the result of an increased risk of severe hypoglycemia due to intensive glucose lowering. Indeed, post hoc analyses from these trials [18, 19] and other studies, e. g., the Outcomes Reduction with an Initial Glargine Intervention (ORIGIN) trial [20], show that severe hypoglycemia, regardless of treatment allocation, is associated with an impaired prognosis. Thus, it is important to avoid hypoglycemia since severe episodes may have a negative impact on survival and mild episodes can affect well-being and treatment inertia. Another potential explanation for the negative results may relate to the side effects of high dosages of glucose-lowering drugs used in different combinations [17, 21].

Reassuringly, a meta-analysis by Ray et al. [22], comprising UKPDS, ADVANCE, ACCORD, VADT, and the Pioglitazone vs. Placebo (PROACTIVE) studies [23], showed that an average reduction in HbA1c levels by 0.9% between patients randomized to intensive compared with conventional glycemic control was associated with a reduction of nonfatal myocardial infarction by 17% (OR: 0.83; 95% CI: 0.75–0.93). It did, however, not affect total mortality (OR: 1.02; 95% CI: 0.87–1.19).

Most international guidelines recommend an individualized glycemic target [21, 24]. The present recommendations for glycemic control (HbA1c ≤ 7%; ≤53 mmol/mol) are mainly based on the prevention of microvascular complications, while evidence for the prevention of CVD is rather weak. For elderly or other fragile patients with long-standing or complicated T2DM, a less strict target (<7.5–8.0%; <58–64 mmol/mol) is safer and thereby sufficient.

### Multifactorial management

The cardiovascular risk is not related only to hyperglycemia. Type 2 diabetes mellitus is a multifactorial disease in which insulin resistance, endothelial dysfunction, and, e. g., factors such as hypertension and dyslipidemia play a contributory role. In fact, about 90% of patients with T2DM are overweight or obese and about 70% have hypertension and/or dyslipidemia [25–27]. In addition to the absolute excess risk for individuals with T2DM, the risk increases with increasing risk factor levels in people with T2DM compared with those without [28]. A multifactorial intervention was tested in the Steno-2 trial where patients with T2DM and microalbuminuria were randomized to intensive, target-driven multifactorial therapy at a specialized clinic or to conventional care [29]. Strict targets were set for lifestyle as well as HbA1c and blood lipid levels, and blood pressure monitoring and treatment with a renin–angiotensin system inhibitor and aspirin were advocated in the intensively managed group. After 7.8 years, there was a 50% reduction in micro- and macrovascular events in this group. Thereafter, target-driven management was recommended for all participants. After 13 years of follow-up, the originally intensively managed patients had an absolute mortality reduction of 20%, and a reduction of cardiovascular events of 29% [30], and after 21 years this reduction translated into 7.9 years of life gained [31], while the risk for hospitalization for heart failure was reduced by 70% [32].

The fundamental importance of multifactorial management of people with T2DM is emphasized in several guidelines, for example, the European Society of Cardiology guidelines on diabetes, pre-diabetes, and CVD from 2013 (update available August 2019; [21]).

### Concerns with glucose-lowering drugs

In a meta-analysis by Nissen and Wolski published in 2007 [33], rosiglitazone—compared with several other glucose-lowering drugs, including metformin, sulfonylureas, and insulin—was associated with a 43% increased risk of myocardial infarction and a 64% increased risk for cardiovascular mortality. This analysis was later criticized because of the inclusion of several studies not representative for the study of cardiovascular outcomes and the negative results were never confirmed in subsequent analyses. Still, the study raised an intensive debate causing rosiglitazone to be withdrawn from the market. Furthermore, the cardiovascular safety of the drug class peroxisome proliferator-activated receptor γ (PPAR-γ) agonists, the thiazolidinediones, was questioned since these drugs were associated with fluid retention, increasing the risk of heart failure in sensitive patients. It was reassuring that the PROACTIVE trial showed that in patients with T2DM and high cardiovascular risk, pioglitazone reduced the secondary endpoint, a composite of death and nonfatal myocardial infarction or stroke (HR: 0.84; 95% CI: 0.72–0.98; *p* = 0.027), while the reduction of the primary endpoint, including leg amputation and revascularization, did not reach statistical significance [23]. Nevertheless, these experiences raised the awareness of, sometimes serious, side effects related to the available glucose-lowering drugs, as listed in Table 1.

**Table 1:** Side effects of various glucose-lowering drugs

Potential problem	Pharmacological agent
Weight gain	Sulfonylureas, glinides, TZDs, insulin
Gastrointestinal	Biguanides, α‑glucosidase inhibitors
Hypoglycemia	Sulfonylureas, glinides, insulin
Lactic acidosis	Biguanides
B_12_ deficiency	Biguanides
Kidney dysfunction	Biguanides, sulfonylureas
Urinary tract infection	SGLT-2 inhibitors
Bladder cancer	TZDs
Hepatic dysfunction	Glinides, TZDs, biguanides
Pancreatitis	DPP-4 inhibitors, GLP-1 agonists
Fractures	TZDs
Cardiovascular concerns	TZDs

*DPP* dipeptidyl peptidase, *GLP* glucagon-like peptide, *SGLT* sodium–glucose cotransporter, *TZDs* thiazolidinediones

At the same time, at the beginning of this century, several new glucose-lowering drug classes were developed and about to be released. Among them were dipeptidyl peptidase-4 (DPP-4) inhibitors, glucagon-like peptide-1 receptor agonists (GLP-1 RA), and sodium–glucose cotransporter-2 (SGLT-2) inhibitors. In 2008, the US Food and Drug Administration (FDA) issued guidance, subsequently also adopted by the European Medicines Agency (EMA), for the industry to ascertain the safety of these new glucose-lowering compounds: “Manufacturers developing new drugs in biologics for type 2 diabetes [are] to provide evidence that the therapy will not increase the risk of such cardiovascular events as a heart attack.” The recommendations resulted in a large number of cardiovascular outcome trials (CVOTs) as outlined in Table 2 and described in more detail in the next section.

**Table 2:** Cardiovascular outcome trials with glucose-lowering drugs (adapted from [40])

Trial [ref.]	Treatment Active/comparator	Patient type (*n*)	Primary endpoint	Follow-up median (years)	Outcome HR (95% CI), *p*	Remarks
*PPAR-γ*						
PROACTIVE [23]	Pioglitazone/placebo	T2DM + macrovascular disease (5238)	A composite of all-cause death, nonfatal MI, stroke, ACS, endovascular or surgical intervention in the coronary or leg arteries, and amputation above the ankle	2.9	No difference 0.90 (0.80–1.02); *p* = 0.095	Secondary outcome composite of CV death and nonfatal MI or stroke 0.84 (0.72–0.98); *p* = 0.027
*Insulin*						
ORIGIN [52]	Insulin glargine/conventional	T2DM, IFG, IGT + high CV risk (12,537)	Composite of CV death and nonfatal MI or stroke	6.2	No difference 1.02 (0.94–1.11); *p* = 0.63	–
DEVOTE [53]	Insulin degludec/insulin glargine	T2DM + CVD, renal disease or high CV risk (7637)	Composite of CV death and nonfatal MI or stroke	1.9	No difference 0.91 (0.78–1.06); *p* < 0.001	–
*SGLT-2 inhibitors*						
EMPA-REG Outcome [39]	Empagliflozin/placebo	T2DM + CVD (7020)	Composite of CV death and nonfatal MI or stroke	3.1	0.86 (0.74–0.99); *p* = 0.0382	Decrease in heart failure hospitalization 0.65 (0.50–0.85) *p* = 0.002
CANVAS [41]	Canagliflozin/placebo	T2DM + high CV risk (10,142)	Composite of CV death and nonfatal MI or stroke	2.4	0.86 (0.75–0.97) *p* = 0.02	Decrease in heart failure hospitalization 0.67 (0.52–0.87) *p* < 0.001
DECLARE-TIMI 58 [43]	Dapagliflozin/placebo	T2DM + CVD or high CV risk (17,160)	Composite of CV death and nonfatal MI or stroke	4.2	0.93 (95% CI, 0.84–1.03) *p* = 0.17	–
*DPP-4 inhibitors*						
TECOS [34]	Sitagliptin/placebo	T2DM + CVD (14,671)	Composite of CV death and nonfatal MI or stroke or hospitalization for UA	3.0	No difference 0.98 (0.89–1.08); *p* = 0.65	No increase in heart failure
EXAMINE [35]	Alogliptin/Placebo	T2DM + recent ACS (5380)	Composite of CV death and nonfatal MI or stroke	1.5	No difference 0.96 (≤1.16); *p* = 0.32	No increase in heart failure
SAVOR-TIMI 53 [36]	Saxagliptin/placebo	T2DM + CVD or high CV risk (16,492)	Composite of CV death and nonfatal MI or stroke	2.1	No difference 1.00 (0.89–1.12); *p* = 0.99	Increase in heart failure hospitalizations in the saxagliptin group
CARMELINA [37]	Linagliptin/placebo	T2DM + high CV or renal risk (6979)	Composite of CV death and nonfatal MI or stroke	2.2	No difference 1.02; 95% CI, 0.89–1.17; *p* < 0.001	No increase in heart failure
*GLP-1 receptor agonists*						
LEADER [45]	Liraglutide/placebo	T2DM + CVD or high CV risk (9340)	Composite of CV death and nonfatal MI or stroke	3.8	0.87 (0.78–0.97); *p* = 0.01	–
SUSTAIN-6 [46]	Semaglutide/placebo	T2DM + CVD, renal disease or high CV risk (2735)	Composite of CV death and nonfatal MI or stroke	1.9	0.74 (0.58 to 0.95); *p* < 0.001	No increase in heart failure
HARMONY-OUTCOMES [48]	Albiglutide/placebo	T2DM + CVD (9463)	Composite of CV death and nonfatal MI or stroke	1.6	0.78 (0.68–0.90) *p* < 0.001	–
REWIND	Dulaglutide/placebo	T2DM + CVD or high CV risk (9622)	Composite of CV death and nonfatal MI or stroke	Not available	Not available	–
ELIXA [50]	Lixisenatide/placebo	T2DM + ACS (6068)	Composite of CV death and nonfatal MI or stroke or hospitalization for UA	2.1	No difference 1.02 (0.89–1.17); *p* = 0.81	No increase in heart failure
EXSCEL [49]	Exenatide/placebo	T2DM +/− CVD (14,752)	Composite of CV death and nonfatal MI or stroke or hospitalization for UA	3.2	No difference	No increase in heart failure

*ACS *acute coronary syndrome, *CI* confidence interval,* CV* cardiovascular, *CVD* CV disease, *DPP* dipeptidyl peptidase, *GLP* glucagon-like peptide, *HR* hazard ratio, *IFG* impaired fasting glucose, *IGT* impaired glucose tolerance, *MI* myocardial infarction, *PPAR-γ* peroxisome proliferator-activated receptor γ, *SGLT* sodium–glucose cotransporter, *TZDs* thiazolidinediones* T2DM* type 2 diabetes mellitus, *UA* unstable angina

### Cardiovascular outcome trials with new glucose-lowering agents

#### DPP-4 inhibitors

The DPP-4 inhibitors are drugs that, by inhibiting its degrading DPP-4 enzyme in a glucose-dependent manner, increase the incretin hormone levels (GLP-1, GIP), thereby increasing the pancreatic endogenous insulin secretion and suppressing glucagon. The DPP-4 inhibitors sitagliptin [34], alogliptin [35], saxagliptin [36], and linagliptin [37] have been studied in large CVOTs (Table 2) in populations of patients with T2DM at high cardiovascular risk. The impact on the primary endpoints (usually a standard major adverse coronary event [MACE] = a composite of cardiovascular death and myocardial infarction or stroke) were neutral in the Saxagliptin Assessment of Vascular Outcomes Recorded in Patients with Diabetes Mellitus (SAVOR) study [36], Examination of Cardiovascular Outcomes with Alogliptin Versus Standard of Care (EXAMINE) study [35], and Cardiovascular and Renal Microvascular Outcome Study With Linagliptin in Patients With Type 2 Diabetes Mellitus (CARMELINA) trial [37]. In the Trial Evaluating Cardiovascular Outcomes with Sitagliptin (TECOS), hospitalization for unstable angina was also included [34]. The lack of improvement in cardiovascular prognosis may be explained by the fact that these trials were designed to shown noninferiority to placebo with short periods of follow-up, i.e., 1.5–3 years. Another explanation may be that DPP-4 inhibitors are merely glucose-lowering agents but without any direct cardiovascular effects.

An unexpected finding in the SAVOR study was that hospitalization for heart failure was significantly more common in patients randomized to saxagliptin than among those allocated to placebo (3.5% vs. 2.8%; HR: 1.27; 95% CI: 1.07–1.51; *p* = 0.007). In the EXAMINE study, the corresponding HR was 1.19 (95% CI: 0.90–1.58; *p* = 0.220). By contrast, no increased risk was seen for sitagliptin in the TECOS study (HR: 1.00; 95% CI: 0.83–1.20; *p* = 0.98; [34]). The reasons for the increased risk of heart failure related to some incretins are not known but it is presumably not a class effect. The FDA issued a warning label in September 2017 for DPP-4 inhibitors regarding the risk of developing heart failure in patients with CVD and without any exception for sitagliptin, which, considering the available evidence, seems unfair.

#### SGLT-2 inhibitors

The SGLT-2 inhibitors increase urinary glucose excretion thereby improving glycemic control in an insulin-independent manner. Besides their glucose-lowering effect, these drugs have the potential to impact the cardiovascular system indirectly, e. g., via weight loss, blood pressure lowering, or directly through osmotic diuresis and increased sodium excretion and presumably also by improving myocardial energetics (increased hematocrit and provision of beta-OH-butyrate [38]).

**Fig. 1:**
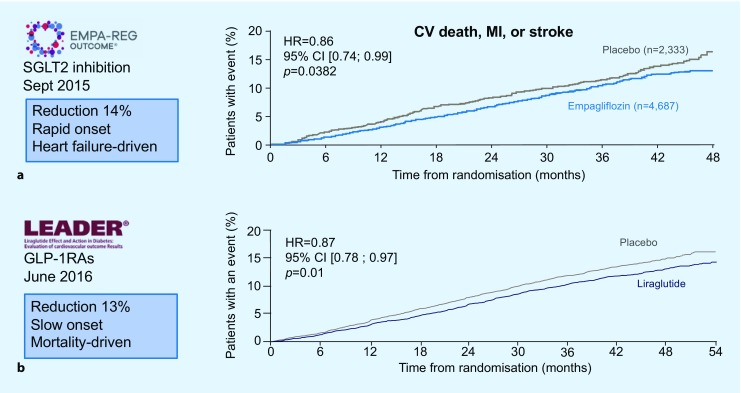
The impact of the sodium–glucose cotransporter 2 (*SGLT2*) inhibitor empagliflozin (**a**) and the glucagon-like peptide 1 (*GLP-1*) receptor agonist liraglutide (**b**) on the primary outcome in the EMPA-REG outcomes and LEADER trials, respectively. Both reduced the rates of cardiovascular death or non-fatal myocardial infarction or stroke to a similar extent (relative reduction 14% and 13%, respectively). This outcome was, however, driven by different event reductions and appeared faster with empagliflozin than with liraglutide. *CI* confidence interval,* CV* cardiovascular, *HR* hazard ratio, *MI* myocardial infarction. (Modified after [39] and [45]. See text for further explanation)

In the Empagliflozin Cardiovascular Outcome Event Trial in Type 2 Diabetes Mellitus Patients-Removing Excess Glucose (EMPA-REG), empagliflozin, compared with placebo, reduced the composite outcome of cardiovascular death or nonfatal myocardial infarction or stroke by 14% (HR: 0.86; 95% CI: 0.74–0.99; *p* = 0.0382) in a patient population (*n* = 7020) with T2DM and established CVD during a median follow-up of 3.1 years (Fig. 1; [39]). In particular, cardiovascular death was reduced, an effect that was seen already within a period of 15 weeks of follow-up, with a substantial reduction of heart failure (HR: 0.65; 95% CI: 0.50–0.85; *p* = 0.0017) as the main driver. These results cannot be explained by the modest reduction in HbA1c levels (−0.24%, compared with placebo), advocating that SGLT-2 inhibitors have important beneficial cardiovascular effects besides glucose lowering [40].

Similar results have been presented for canagliflozin in the Canagliflozin Cardiovascular Assessment Study (CANVAS) program in patients with a mean duration of diabetes of 13 5 years and 65% with a history of CVD. The improvement in cardiovascular events amounted to 24% (HR: 0.86; 95% CI: 0.75–0.97; *p* < 0.001 for noninferiority; *p* = 0.02 for superiority; [41]). However, in addition to the known adverse events with SGLT-2 inhibitors, most importantly genital infections, the CANVAS program reported on a small but significant increased risk of lower limb amputations. There was also a small increase in bone fractures but this finding was not confirmed in a large database investigation in which 79,964 patients initiated on canagliflozin were identified and matched to 79,964 patients initiated on a GLP-1 agonist (HR: 0.98; 95% CI: 0.75–1.26; [42]).

In the recently published Dapagliflozin Effect on Cardio-vascular Events–Thrombolysis in Myocardial Infarction 58 (DECLARE-TIMI 58) trial [43], for 17,160 patients with T2DM who had or were at risk (*n* = 10,186) of atherosclerotic CVD, treatment with dapagliflozin did not result in a higher or lower rate of major cardiovascular events than placebo but in a lower rate of cardiovascular deaths or hospitalization for heart failure.

In addition to the effects on the cardiovascular system, in particular related to heart failure, the three aforementioned SGLT-2 inhibitors were associated with improvements in the renal endpoints in the respective CVOT.

#### GLP-1 receptor agonists

The GLP-1 RAs improve glycemic control via the incretin hormone system. They are associated with improvements in several cardiovascular risk factors, among them body weight, blood pressure, and lipid levels. The GLP-1 receptors are widely distributed in the body. Through their impact on these factors it is reasonable to assume that GLP-1 RAs have pleiotropic effects such as a reduction of inflammatory activation and atherosclerosis progress, renal protection, and potentially even direct myocardioprotection [44].

In the Liraglutide Effect and Action in Diabetes: Evaluation of Cardiovascular Out-come Results (LEADER) trial, liraglutide was superior to placebo in reducing the primary composite MACE outcome (HR: 0.87; 95% CI: 0.78–0.97; *p* = 0.01; Fig. 1). The absolute reduction (ARR) amounted to 1.9% over 3.8 years and included a decrease of all-cause mortality (HR: 0.85; 95% CI: 0.74–0.97; *p* = 0.02; ARR 1.4%; [45]). Similar results were shown in the pre-marketing Trial to Evaluate Cardiovascular and Other Long-term Outcomes with Semaglutide in Subjects with Type 2 Diabetes (SUSTAIN-6), where semaglutide reduced MACE (HR: 0.74; 95% CI: 0.58–0.95; *p* = 0.02 for superiority). The ARR was 2.3% during 2.1 years in 3297 patients with T2DM and established or high risk for CVD [46]. The reduction in events was mainly driven by the rate of stroke rather than CVD death as in the LEADER study.

An unexpected finding was an increase in retinopathy, which in a sub hoc analysis was shown to mainly occur in those with pre-existing eye problems and presumably associated with the rapid reduction in glucose. A subsequent US population-based cohort study of older adults with diabetes suggests that incretin-based therapies, including GLP-1 RA, used for approximately 1 year, did not increase the retinopathy risk [47].

Recently, albiglutide was shown to be superior to placebo in the Harmony Outcomes in Reducing Cardiovascular Events (HARMONY Outcomes) trial (*p* < 0.0001 for noninferiority; *p* = 0.0006 for superiority; [48]) and a press release from the REWIND study indicates similar results for dulaglutide.

Interestingly, the beneficial effects on cardiovascular events of GLP-1 RA did not, in contrast to the SGLT-2 inhibitors, occur during the first phase of the trials. Taken together with animal and some small human studies, this suggests that it may be a result of improvements in inflammation and a retardation of the atherosclerotic process [44].

In contrast to the aforementioned trials, in the EXSCEL [49] and ELIXA studies [50], exenatide extended-release and lixisenatide were not inferior but also not superior to placebo in preventing cardiovascular events. Several potential explanations for the discrepant findings have been suggested. They may, for example, be related to differences in patient populations. All participants in the ELIXA trial had, in addition to T2DM, a recent acute coronary syndrome, and the proportion of patients at high risk for or already established CVD differed between the various trials. Other reasons may relate to the different drugs and molecules and to the different molecular size and biological half-life, particularly if the drugs are based on the synthetic exendin-4 (e. g., exenatide and lixisenatide) or on human GLP-1 analogues (liraglutide, semaglutide, dulaglutide, and albiglutide [44]).

### New versus old glucose-lowering drugs

When it comes to older classes of glucose-lowering drugs such as sulfonylureas, the impact on cardiovascular events has not been systematically investigated in randomized trials comparing them with DPP-4 inhibitors, GLP-1 RAs, or SGLT-2 inhibitors. However, in a Swedish registry-based study, 77% of 52,760 patients who had been prescribed sulfonylurea as add-on glucose-lowering therapy to metformin were compared with the 23% of patients who instead had received a DPP-4 inhibitor. The crude incidence of all-cause mortality in the sulfonylurea cohort was 24.6/1000 patient-years compared with 14.9/1000 patient-years in the DPP-4 cohort. Sulfonylurea compared with DPP-4 inhibition was associated with a higher risk of subsequent severe hypoglycemia, fatal and nonfatal cardiovascular disease, and all-cause mortality (adjusted HR: 2.07, 95% CI: 1.11–3.86; HR: 1.17, 95% CI: 1.01–1.37; and HR: 1.25, 95% CI: 1.02–1.54, respectively; [51]). A potential explanation may be the higher risk for prolonged hypoglycemia, in particular in vulnerable patients.

Data on exogenous insulin in this setting are also sparse. However, one of the first large CVOTs was ORIGIN, comparing insulin glargine with conventional glucose-lowering treatment in patients with T2DM, impaired glucose intolerance, and impaired fasting glucose at high cardiovascular risk. The hypothesis that early institution of basal insulin would improve the prognosis was, however, not confirmed as the effect was neutral between the randomized treatment groups [52]. In the DEVOTE trial, insulin degludec and glargine were comparable with respect to the incidence of major cardiovascular events [53].

### Interventions against insulin resistance

The recent Insulin Resistance Intervention After Stroke (IRIS) study included 3876 patients free from type T2DM but insulin resistant according to the homeostasis model assessment of insulin resistance index (HOMA-IR) and with a recent ischemic stroke or transient ischemic attack. They were randomized to either pioglitazone or placebo. After 4.8 years of follow-up, 9.0% of the pioglitazone-treated patients compared with 11.8% in the placebo group had experienced the primary outcome, a composite of fatal or nonfatal stroke or myocardial infarction (HR: 0.76; 95% CI: 0.62–0.93; *p* = 0.007; [54]). Diabetes developed in 3.8% and 7.7% of the pioglitazone- and placebo-treated patients, respectively. These trials suggest that improving insulin sensitivity, one of the main effects of the (PPAR-γ) agonists, may not only reduce cardiovascular complications in patients with established diabetes but also in those in a pre-disease state.

## Gaps in knowledge

The reasons why SGLT-2 inhibitors and GLP-1 receptors are associated with improvements in cardiovascular benefits are not fully understood. Further mechanistic studies are needed to clarify whether the beneficial effects relate to improvements in the cardiovascular risk factor profile or to pleiotropic cardiovascular effects, and the results of such studies need clinical verification. The CVOTs with SGLT-2 inhibitors suggest that the beneficial effects of these drugs are mediated by a decrease in heart failure. It should, however, be noted that these studies did not primarily study patients with heart failure or such events as primary outcomes. Trials with SGLT-2 inhibitors in patients with established heart failure, with or without diabetes, are ongoing and will hopefully shed further light. This is important, not least because heart failure is an important complication to T2DM as already underlined. GLP-1 RA may, as discussed, have an impact on the process of atherosclerosis.

Another important aspect is whether the beneficial effects of GLP-1 RA and SGLT-2 inhibitors are present in people without established CVD, early in the course of dysglycemia, e. g., the insulin-resistant state and in pre-diabetes. Some studies, e. g., LEADER and REWIND, recruited patients with cardiovascular risk factors but free from established CVD, but it is still difficult to extrapolate the results to a primary preventive setting. To establish whether the drugs work in these states is of great interest mainly because there are indications from meta-analyses that people with a shorter duration of dysglycemia (<5 years from onset of diabetes) benefit more from intensive glucose control, compared with treatment starting later or treatment in patients who have already developed signs of vascular damage [55].

Finally, the optimal glycemic target from a cardiovascular perspective remains to be defined. It could be that with the newer classes of drugs, with less risk of hypoglycemia in addition to their established cardiovascular effects, a more intensive glucose lowering may have added value.

## Conclusion

An important task for the diabetology, cardiology, as well as the general practice communities is to incorporate the findings of the CVOTs into clinical practice. In a consensus report by the American Diabetes Association (ADA) and the European Association for the Study of Diabetes (EASD), it is stated that the presence of CVD and/or chronic kidney disease should be assessed. After the first-line therapies with metformin and comprehensive lifestyle interventions, SGLT-2 inhibitors are recommended in people with heart failure and GLP-1 RAs in those with atherosclerotic CVD.
